# The effect of patient positioning during radiographs on the resulting Cobb angle measurements

**DOI:** 10.1186/1748-7161-7-S1-O12

**Published:** 2012-01-27

**Authors:** M Siljander, P Knott, S Thompson, S Mardjetko

**Affiliations:** 1 Rosalind Franklin University North Chicago, USA; 2 Illinois Bone and Joint Institute Morton Grove, USA

## Background

Standing spinal radiographs have been the primary method of spinal deformity evaluation in patients with scoliosis. During periods of patient surveillance, the clinician compares radiographs over a period of time to assess the progression of the deformity [1-3]. One of the potential problems in comparing one radiograph to another is difference in positioning [4-6]. The goal of this study is to quantify the effect of trunk rotation on Cobb angle measurements, and provide an algorithm to describe this relationship.

## Material and methods

CT scans of three patients with Adolescent Idiopathic Scoliosis were used retrospectively. Three-dimensional reconstructions of the images were created by CT scan software. Cobb angles were drawn for scoliosis curves in the anterior plane. The 3-D image was then rotated two degrees to the right, and Cobb angle measurements were repeated. This procedure was repeated through 14 degrees of right rotation, and then subsequently through 14 degrees of left rotation.

## Results

The effect of trunk rotation on Cobb angle measurements is directly related to the location of the scoliosis curves, the magnitude of those curves, and the magnitude of lumbar lordosis and thoracic kyphosis. In general however, a two degree rotation of the patient’s trunk while positioning results in a one degree change in the measured Cobb angle (in patients with larger scoliosis curves, and in the first six degrees of trunk rotation).

## Conclusions

Patient positioning can have a significant effect on the calculation of scoliosis measurements, and this needs to be considered when evaluating the progression of spinal deformity.
